# Evaluation of Pharmacogenetics of Drug-Metabolizing Enzymes and Drug Efflux Transporter in Renal Transplants Receiving Immunosuppressants

**DOI:** 10.3390/jpm12050823

**Published:** 2022-05-19

**Authors:** Kannan Sridharan, Shamik Shah, Anfal Jassim, Mona Hammad, Johaina Ebrahim Al Gadhban, Ola Al Segai

**Affiliations:** 1 Department of Pharmacology & Therapeutics, College of Medicine & Medical Sciences, Arabian Gulf University, Manama 328, Bahrain; 2 Department of Nephrology, Salmaniya Medical Complex, Manama 328, Bahrain; shamik.shah@gmail.com (S.S.); jghadhban@health.gov.bh (J.E.A.G.); 3 Department of Internal Medicine, College of Medicine & Medical Sciences, Arabian Gulf University, Manama 328, Bahrain; 4 Department of Molecular Medicine, College of Medicine and Medical Sciences, Arabian Gulf University, Manama 328, Bahrain; anfalaaj@agu.edu.bh; 5 Salmaniya Medical Complex, Manama 328, Bahrain; mona.hammad717@gmail.com; 6 Department of Biochemistry, Salmaniya Medical Complex, Manama 328, Bahrain; osegai@health.gov.bh

**Keywords:** sirolimus, tacrolimus, cyclosporine

## Abstract

Cytochrome P450 (CYP) enzymes, such as *CYP3A4,* and *CYP3A5*, P450 oxidoreductase (POR), peroxisome proliferator activated receptor alpha (PPAR-alpha), and drug transporter (ABCB1) were observed to influence concentrations of immunosuppressants (cyclosporine, everolimus, sirolimus, and tacrolimus) and outcomes in renal transplants. We carried out the present study to evaluate the prevalence and impact of these single nucleotide polymorphisms (SNPs) in adult renal transplants. SNPs were evaluated using commercial TaqMan^®^ assays. Serum drug concentrations were estimated using immunoassays. One hundred and forty-six patients were recruited. SNPs in CYP3A5*3 were significantly associated with greater dose-adjusted cyclosporine and tacrolimus concentrations. SNPs in POR*28 were observed with significantly lower dose-adjusted concentrations, particularly with cyclosporine and tacrolimus. *ABCB1* homozygous polymorphisms were observed with significantly lower time spent in the therapeutic range with cyclosporine and everolimus/sirolimus. Cyclosporine was observed in a significantly greater proportion of patients with elevated GGT, and SNPs in *PPAR-alpha* were significantly associated with an increased risk of this adverse event. Hypertriglyceridemia with everolimus was significantly associated with POR*28 polymorphisms. There is a need to validate the influence of these SNPs in a prospective study and develop an algorithm predicting the achievement of target concentrations.

## 1. Introduction

Immunosuppressive drugs, such as cyclosporine, sirolimus, and tacrolimus, form the mainstay of drugs often administered lifelong to patients with renal transplantation [1]. Of these, tacrolimus is the most preferred due to its greater potency, and lesser risks of rejection and nephrotoxicity [2]. Wide inter-and intra-patient variability, narrow therapeutic window, and the risk of toxicity are the key factors driving therapeutic drug monitoring of these immunosuppressants [3]. Maintaining drug concentrations in the therapeutic range is crucial for preventing rejection episodes and toxicity amelioration [4]. 

Cytochrome P450 (CYP) enzymes, predominantly *CYP3A4* and *CYP3A5,* are involved in the metabolism of the calcineurin inhibitor class of immunosuppressants [5]. *CYP3A4* is the most important metabolizing enzyme, considering the total number of drugs that undergo biotransformation. Amongst this family, the most recognized are CYP3A4*1B, *2, and *3, and, recently, CYP3A4*22 has been observed to play a significant role in the expression of CYP3A4 enzymes [6,7]. CYP3A4*22 contributes to 12% of *CYP3A4* enzymatic variability [8]. Regarding the *CYP3A5* family, 11 isoforms have been identified of which CYP3A5*3 is the most important, due to its functional significance [9]. CYP3A5*3, particularly in its homozygous form, results in the formation of truncated non-functional proteins resulting in absent *CYP3A5* enzymatic activity [10]. Calcineurin inhibitors are also substrates of a drug transport protein, namely, P-glycoprotein (ABCB1), in the intestines, and renal tubules. Several polymorphisms have been identified in the *ABCB1* gene influencing the activity of the efflux transporter [11]. The three most common single nucleotide polymorphisms (SNPs) identified with *ABCB1* transporter include 1236C > T, 2677G > T, 3435C > T, and 1199G > A [12]. These SNPs have been observed with decreased efflux activity of *ABCB1* transporter of tacrolimus and sirolimus [13]. More recently, peroxisome-proliferator activated receptor alpha (PPAR-alpha) has been noted as it exhibits influence on the expression of *CYP3A4* enzymes, and SNP (rs4253728) contributes to around 8–9% of its enzymatic activity [14]. Similarly, Cytochrome P450 oxidoreductase (POR) is a microsomal flavoprotein forming an important component of CYP enzymes [15]. POR*28 polymorphism has been associated with significantly lower CYP3A activity [16]. The complex interplay of these SNPs on the therapeutic concentrations of cyclosporine, tacrolimus, sirolimus, and everolimus has hardly been systematically explored. Hence, we undertook the present study to evaluate the associations between the above-mentioned SNPs with serum concentrations of the immunosuppressive drugs administered in renal transplant patients in the Bahraini population.

## 2. Materials and Methods

### 2.1. Study Design and Ethics

This was a cross-sectional study carried out between April 2021 and February 2022 after obtaining approval from the Institutional Ethics Committee. Written consent was obtained from the study participants. The latest Declaration of Helsinki guidelines was adhered to. 

### 2.2. Study Procedure

Adult (>21 years) renal transplant patients of either sex that were being followed up in the Department of Nephrology, Salmaniya Medical Complex, Kingdom of Bahrain, were recruited following their consent. Only those receiving any of the following immunosuppressant drugs for at least one year were recruited: tacrolimus, everolimus, sirolimus, and cyclosporine. We obtained the details on their demographics (age and gender), duration of renal transplantation, laboratory parameters (such as creatinine, estimated glomerular filtration rate, plasma concentrations of the immunosuppressants, serum cholesterol, and liver function tests), drug-related details (names, dose, frequency, and route) and the serum concentration/s of any of the above-mentioned immunosuppressants that were carried out as a part of their standard of care. 

### 2.3. Estimation of Genetic Polymorphisms

Two milliliters of blood were collected for evaluating the following SNPs: CYP3A4*22 (rs35599367), CYP3A5*3 (rs776746), *ABCB1 1236 C* > *T* (rs1128503), *PPAR-alpha* (rs4253728), and POR*28 (rs1057868). The genomic DNA was extracted from the peripheral blood leukocytes using a QIAamp^®^ DNA blood mini kit (Qiagen). The concentrations of DNA were measured using a Nanodrop spectrophotometer [17]. SNPs were genotyped using the allelic discrimination method on a StepOne Plus^®^ real-time PCR system (Applied Biosystems; Foster City, CA, USA) according to the manufacturer’s instructions using commercially available TaqMan^®^ assays. The following were the categories of genotypes considered for each of the SNPs: CYP3A4*22 (GG-wild; GA-heterozygous; and AA-homozygous), CYP3A5*3 (TT-wild; CT-heterozygous; and CC-homozygous), *ABCB1* (AA-wild; AG-heterozygous; and GG-homozygous), *PPAR-alpha* (AA homozygous; GG homozygous; and AG-heterozygous), POR*28 (CC-wild; CT-heterozygous; and TT-homozygous). POR*28 activity was considered normal with CC and CT genotypes and higher with TT genotype [18].

### 2.4. Estimation of Blood Tacrolimus, Sirolimus, and Cyclosporine Concentrations

Whole blood concentrations of cyclosporine, tacrolimus, sirolimus, and everolimus were estimated using chemiluminescent microparticle immunoassay. The assay was an automated two-step immunoassay, wherein the whole blood sample was lysed with a solubilization agent, extracted with a precipitation reagent, and centrifuged. The resulting supernatant was decanted into a pretreatment tube. Anti-drug coated microparticles, the pretreated sample, and assay diluents were then mixed and incubated, followed by washing. Then acridinium-labeled conjugates, specific to the immunosuppressive drugs, were added, following which the chemiluminescent reaction was measured as a relative light unit. The assay linearity ranged between 18 and 1500 ng/mL, the limit of detection was 6.7 ng/mL, and the limit of quantification was 18 ng/mL for cyclosporine. The limit of quantification ranged between 2 and 30 ng/mL for tacrolimus, sirolimus, and everolimus. 

Blood samples for estimation of drug concentrations were carried out just before the morning dose. Concentrations in the above-mentioned ranges were considered therapeutic; below the lower limit was considered sub-therapeutic, and above the upper limit of the reference range supra-therapeutic. Dose-adjusted concentrations were obtained by dividing the concentrations by the daily dose for each immunosuppressive drug. Percent time spent in the therapeutic range was calculated by the linear interpolation method. A linear movement of the serum concentrations of immunosuppressive drugs is assumed and a value is assigned every day between the two consecutive laboratory values. Finally, the percent time spent in the therapeutic range for each immunosuppressive drug was estimated from the total duration.

### 2.5. Laboratory Reference Ranges

Reference ranges in our laboratory for serum cyclosporine were 90–150 ng/mL, sirolimus and everolimus were 3–20 ng/mL, and tacrolimus was 5–20 ng/mL. There is no consensus on the established therapeutic ranges for immunosuppressants and, considering the observed therapeutic and adverse effects in our population, the above-mentioned ranges are being followed by our hospital laboratory as a part of the standard of care. The reference ranges for the serum biochemical parameters are as follows: creatinine: 53–97 µmol/L; total cholesterol: 3.6–5.2 mmol/L; triglycerides: 0.2–1.8 mmol/L; low-density lipoprotein (LDL) cholesterol: 1.7–3.4 mmol/L; total bilirubin: 5–21 µmol/L; alkaline phosphatase (ALP): 50–136 U/L; alanine aminotransferase (ALT): <41 U/L; and Gamma-glutamyl transferase (GGT): 15–85 U/L. Serum creatinine elevation >1.5 times the upper limit of the normal range (ULN) was considered significant. Similarly, elevations in the serum ALP and ALT were considered significant if they were at least 5-fold the ULN. Elevations above the ULN were considered significant for all other biochemical parameters. 

### 2.6. Statistical Analysis

Descriptive statistics were used for representing the demographic variables. Kruskal-Wallis H test was used for comparing the number of samples in the sub-therapeutic, therapeutic, and supra-therapeutic categories between the immunosuppressive drugs. Mann-Whitney U test was used for analyses of differences in the concentrations between normal and higher POR*28 activity. Bonferroni corrected *p*-values were considered for statistical significance. Jonckheere-Terpstra test was used for trend analysis of the numerical variables. Multiple linear regression analyses were carried out with the dose-adjusted drug concentrations as the dependent variable and SNPs as the independent variables. Regression coefficients (β) with 95% confidence intervals (CI) were used for representing the change in the dose-adjusted concentrations with the SNPs. Multinomial logistic regression analyses were carried out with the predominant category of serum concentrations (sub-therapeutic, therapeutic, and supratherapeutic) as the dependent variable and the evaluated SNPs as the independent variables. Odds ratio (OR) with 95% CI was used as the measure of effect estimate in the logistic regression analysis. Chi-square test for association was used for analysis of association between the SNPs and laboratory-related adverse events along with OR. A *p*-value of ≤0.05 was considered statistically significant. SPSS version 28 (IBM Corp. Released 2020. IBM SPSS Statistics for Windows, Version 27.0. Armonk, NY: IBM Corp.) was used for statistical analyses. 

## 3. Results

### 3.1. Demographics

One hundred and forty-six patients were recruited, and a summary of their key demographic characteristics is listed in Table 1. 

### 3.2. Immunosuppressive Drugs and Serum Levels

Seventy-six (52.1%) were receiving tacrolimus, 43 (29.5%) cyclosporine, 22 (15.1%) everolimus, three (2.1%) sirolimus, and one each (1.2%) received everolimus along with tacrolimus, and everolimus along with cyclosporine. The dosing regimens received by the study participants are listed in Table 2.

**Figure 1:**
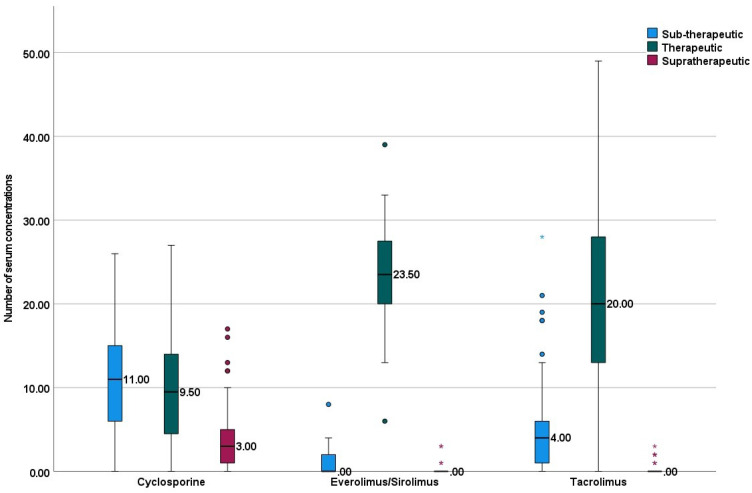
Comparison of categories of drug concentrations.

**Figure 2:**
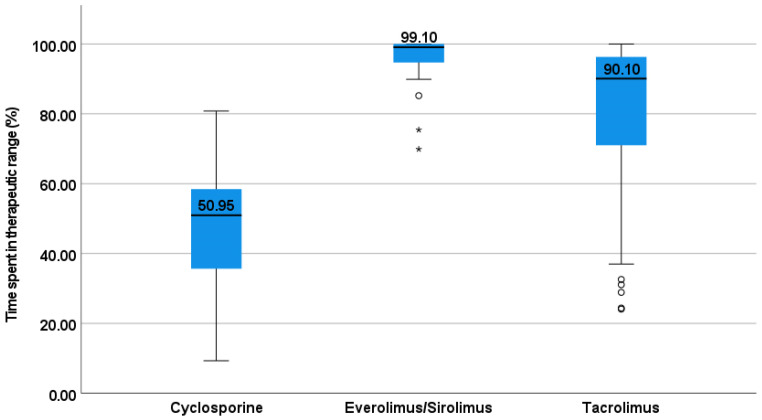
Comparison of time spent in the therapeutic range.

Median (range) of serum cyclosporine was 97 (19–592.5) ng/mL, everolimus/sirolimus was 5.2 (2–27.6) ng/mL, and tacrolimus was 7.1 (2–30) ng/mL. Median (range) number of concentrations in the sub-therapeutic range was significantly higher with cyclosporine {11 (0–26)} compared to tacrolimus {4 (0–28)}, and everolimus/sirolimus {0 (0–8)} (Figure 1). Median (range) time spent in therapeutic range 84 (24.1–100). Cyclosporine was observed with the least time spent in the therapeutic range; this was statistically significant, compared to other immunosuppressive drugs (Figure 2). 

This cluster boxplot depicts distributions of number of concentrations in sub-therapeutic, therapeutic, and supratherapeutic categories. The horizontal lines in the boxplots represent the median and the vertical lines indicate the ranges. Stars and circles represent the outliers. 

This simple box plot depicts the time spent in therapeutic range wherein the upper and the lower horizontal lines on the blue boxes represent the 25th and 75th percentile respectively, with the horizontal black line indicating the median values. Stars and circles represent the outliers. 

Median (range) of mean drug concentrations for cyclosporine was 103 (69.9–333.5) ng/mL, everolimus/sirolimus was 5.5 (2–12.1) ng/mL, and tacrolimus was 7.6 (3.8–16.5) ng/mL. Similarly, the median (range) dose-adjusted drug concentrations for cyclosporine were 1 (0.3–4.6) ng/mL/mg, everolimus/sirolimus was 5 (2–23.8) ng/mL/mg, and tacrolimus was 3.5 (0.8–19.7) ng/mL/mg.

### 3.3. Prevalence of SNPs

The minor allele frequencies in CYP3A4*22, CYP3A5*3, *ABCB1 1236 C* > *T*, *PPAR-alpha,* and POR*28 were 0.01, 0.38, 0.42, 0.11, and 0.29 respectively. The predominant genotypes in *ABCB1* were AG (80, 54.8%), POR*28 was CC (66, 45.2%), *PPAR-alpha* was GG (94, 64.4%), CYP3A4*22 was GG (120, 82.2%), and CYP3A5*3 was CC (92, 63.1%) (Table 3). Eighteen (12.3%) had higher POR*28 activity with a normal activity in the remaining.

### 3.4. Association between Drug Concentrations and SNPs

Median (range) dose-adjusted concentrations in the overall study population according to the evaluated SNPs are listed in Table 4. A significantly lower dose-adjusted concentration was observed with heterozygous SNPs in POR*28. The median (range) concentrations and dose-adjusted concentrations for the individual immunosuppressive drugs are mentioned in Table 5 and Table 6, respectively. *ABCB1* polymorphisms significantly influence the serum tacrolimus concentrations. SNPs in POR*28 were associated with significantly lower dose-adjusted concentrations with cyclosporine (both homozygous and heterozygous), and tacrolimus (heterozygous). Similarly, homozygous CYP3A5*3 SNPs were associated with greater dose-adjusted tacrolimus concentrations. Multiple linear regression analyses revealed significant associations between POR*28 SNPs with dose-adjusted cyclosporine concentrations (β: −0.3; 95% CI: −1, −0.05; *p* = 0.03). 

### 3.5. Association of SNPs with the Time Spent in the Therapeutic Range

Time spent in the therapeutic range for the immunosuppressive drugs with the evaluated SNPs is summarized in Table 7. GG genotype in *ABCB1* was observed with significantly lower time spent in the therapeutic range with cyclosporine and everolimus/sirolimus.

### 3.6. Association of SNPs with the Laboratory Adverse Events

Table 8 summarizes the incidences of laboratory-related adverse events amongst the study participants. A significant proportion of patients with GGT elevation was observed with cyclosporine compared to other drugs. None of the patients had any significant changes in serum ALP and one each with cyclosporine and everolimus/sirolimus, and two receiving tacrolimus had elevated ALT. Evaluation of the associations between the genotypes and the changes in the laboratory parameters revealed a significant association between SNPs in *PPAR-alpha* and GGT elevation with cyclosporine (OR: 0.2, 95% CI: 0.04, 0.6; *p* = 0.01). Also, a significant association between POR*28 polymorphism with hypertriglyceridemia was observed with everolimus/sirolimus (OR: 0.1, 95% CI: 0.01, 1; *p* = 0.05). 

## 4. Discussion

### 4.1. Key Findings from the Present Study

We evaluated the associations of key SNPs with serum drug concentrations of cyclosporine, sirolimus, and tacrolimus in 146 patients with renal transplantation. The time spent in the therapeutic range was maximum with everolimus/sirolimus, followed by tacrolimus. The predominant genotypes in *ABCB1* were AG (54.8%), POR*28 was CC (45.2%), *PPAR-alpha* was GG (64.4%), CYP3A4*22 was GG (82.2%), and CYP3A5*3 was CC (63.1%). SNPs in CYP3A5*3 were associated with significantly greater dose-adjusted tacrolimus concentrations. Additionally, SNPs in POR*28 were observed with significantly lower dose-adjusted cyclosporine and tacrolimus concentrations. Homozygous *ABCB1* homozygous polymorphisms were observed with significantly lower time spent in the therapeutic range with cyclosporine and everolimus/sirolimus. Cyclosporine was observed with a significantly greater proportion of patients with elevated GGT, and the SNPs in *PPAR-alpha* were associated with a significantly increased risk of this adverse event. Hypertriglyceridemia with everolimus was significantly determined by POR*28 polymorphisms. 

### 4.2. Comparison with the Existing Literature

We observed that nearly two-thirds of the Bahraini population carried homozygous CYP3A5*3. Similar frequencies of CYP3A5*3 were observed in Europeans (94%) and admixed Americans (80%) [19]. A recent study from the Qatari population estimated the presence of intermediate metabolizer status with the *CYP3A5* family to an extent of 16.5% [20]. The presence of homozygous CYP3A5*3 has been associated with reduced enzymatic activity to the extent of requiring only 50% of the tacrolimus dose [21]. In the present study, we also observed significantly greater dose-adjusted tacrolimus concentrations with CYP3A5*3 alleles. Those with heterozygous CYP3A5*3 had 50% greater dose-adjusted tacrolimus concentrations and those with homozygous alleles had 135% greater dose-adjusted tacrolimus concentrations in the present study. CYP3A5*3 was observed with reduced clearance of tacrolimus (heterozygous—0.8 L/h/kg; and homozygous—0.5 L/h/kg) compared to wild genotypes (1 L/h/kg) [22]. Studies have observed higher dosing requirements to an extent of around 1.5 times with CYP3A5*3 carriers [23]. Another related family of CYP enzymes involved in the metabolism of immunosuppressive drugs is *CYP3A4*. The prevalence of CYP3A4*22 was observed to an extent of 5% in the European population and 3% in admixed American populations [19]. We observed a slightly higher prevalence (8.9%) in the Bahraini population. CYP3A4*22 was identified with reduced dose-adjusted concentrations of tacrolimus and, subsequently, with reduced dosing requirement [24]. Moes et al. observed that CYP3A4*22 carriers had 15, 7 and 16% reduced clearances for cyclosporine, everolimus, and tacrolimus [25]. We did not observe any significant association with CYP3A4*22 with the dose-adjusted concentrations, but a significantly lower risk of achieving sub-therapeutic concentrations was observed with everolimus/sirolimus. Although a higher area-under-the-concentration-time curve was observed with tacrolimus with mainly CYP3A5*3 and, to a lesser extent, CYP3A4*22, no clinically significant differences were observed in terms of rejection episodes [26]. *CYP3A5* genotype-based tacrolimus dosing was observed to result in the earlier achievement of therapeutic concentrations compared to the standard of care in a randomized clinical trial [27]. However, another randomized clinical trial did not observe any significant differences in terms of time required to achieve therapeutic concentrations, the proportion of patients with sub- or supra-therapeutic concentrations, and acute rejection episodes between *CYP3A5* genotype-based tacrolimus dosing and the standard dosing regimen [28]. Although the *CYP3A5* genotype is the strongest predictor of tacrolimus dose, two-thirds of the variability is explained by other factors. 

We observed a minor allele frequency in POR*28 to an extent of 29%. This consisted of African Americans (20%), Caucasians (28.6%), and Asians (38.9%) [29]. POR*28 activity has been observed to influence the serum concentrations of immunosuppressive drugs, particularly amongst those with *CYP3A5* non-expression [30]. A reduction of 24% in the tacrolimus and 15% with cyclosporine dose-adjusted concentrations were observed amongst those with POR*28 carriers [30]. We observed a reduction of around 27% with cyclosporine, and an 18.5% reduction with tacrolimus dose-adjusted concentrations amongst the carriers of POR*28. A recent meta-analysis also concluded that POR*28 carriers showed a mean difference of 8.3 ng/mL per mg/kg tacrolimus concentrations compared to the wild genotype [31]. The only other study that has evaluated the influence of POR*28 on sirolimus concentrations was that by Woillard et al., where the authors observed a significant, but minor, reduction in serum concentrations amongst carriers [32]. However, the authors also observed that there was no need for any dosage modification for sirolimus. In the present study, we did not observe any significant difference in the dose-adjusted concentrations for sirolimus, but carriers of POR*28 were observed to have an increased risk of having sub-therapeutic concentrations. Apart from the metabolizing enzymes, SNPs influence the rate and extent of drug absorption by the most important family of efflux transporters, *ABCB1*. *ABCB1* 1236 C > T was observed with a frequency of around 42% in the present study. Studies report a prevalence ranging between 13% among African Americans and 62% amongst European Americans [33]. *ABCB1* polymorphisms result in reduced efflux of immunosuppressive drugs, both from the intestine (resulting in better bioavailability) and in the hepatocytes (resulting in reduced elimination) [34]. Hence, their impact on the serum concentrations of drugs effluxed by these transporters is variable. A recent meta-analysis confirmed a reduced dosing requirement for sirolimus in patients with homozygous mutants of *ABCB1* 1236 C > T [35]. On the contrary, Llaudo et al. did not observe any significant impact of the p-glycoprotein polymorphisms on either sirolimus or tacrolimus but was negatively correlated with cyclosporine concentrations [36]. Similar haplotypes in ABCB1 were not observed to significantly predict the initial tacrolimus concentrations [37]. Although we did not observe any significant difference in the dose-adjusted concentrations of the immunosuppressive drugs with *ABCB1* polymorphisms, individuals with homozygous mutants were observed with significantly lower time spent in the therapeutic range. This may be one of the reasons why *ABCB1* 1236 C > T has been observed with an increased risk of acute rejection in a previous study [38]. More studies delineating the roles of ABCB1 polymorphisms are needed to understand their clinical utility. 

Cyclosporine has been reported to cause biliary sludge and cholelithiasis [39]. Individuals with cyclosporine-induced severe liver injury were observed with elevated GGT [40]. In the present study, we also observed that a significantly greater number of patients on cyclosporine had elevated GGT, compared to other immunosuppressive drugs. Similarly, hypertriglyceridemia is a commonly observed adverse event with all the immunosuppressants, particularly more so with everolimus, due to its effect of modulation on the expression of lipoprotein lipase [41]. The existing literature did not aid in identifying a direct link between the *PPAR-alpha* with GGT elevation, and POR*28 with hypertriglyceridemia. Future studies are warranted to explore these possible associations. 

### 4.3. Strengths and Limitations

This is the first comprehensive study evaluating the influence of five genetic polymorphisms on serum concentrations and laboratory-related adverse events in the Middle Eastern population, specifically in Bahrainis. Also, to the best of our knowledge, this is the first study incorporating an outcome related to time spent in the therapeutic range for immunosuppressive drugs. The importance of this outcome can best be understood by the critical attributes of immunosuppressants, such as narrow therapeutic window and increased risk of toxicity profile. There could be variations in the recommended therapeutic ranges between the immunosuppressive drugs between the institutions. We adhered to the reference ranges practiced in our hospital and we recommend the readers interpret the results with this background understanding. Secondly, we did not attempt to calculate the sample size a priori, but post-hoc calculations revealed a power ranging between 71.1 and 74% for the primary outcome (association of SNPs in CYP3A5*3 with tacrolimus). Clinical outcomes, such as rejection episodes and adverse events, could not be captured. 

## 5. Conclusions

We observed significant influences of certain SNPs on serum concentrations and laboratory-related adverse events with immunosuppressive drugs in the Bahraini population. A significantly greater dose-adjusted tacrolimus concentration was observed with CYP3A5*3 polymorphisms and dosage modification should be considered in this population. SNPs in POR*28 and *ABCB1* were observed to influence parameters related to sub-therapeutic concentrations and time spent in the therapeutic range. There is a need to validate the influence of these SNPs in a prospective study and to develop an algorithm predicting the achievement of target concentrations.

## Figures and Tables

**Table 1:** Demographics of study participants (N = 146).

Parameters		Values
Age (years) ^$^		50.5 (21–74)
Males: Females (n)		90: 56
Duration of renal transplantation (years)		7 (1–34)
Immunosuppressive drugs (n) ^#^	Tacrolimus	77
	Cyclosporine	44
	Everolimus	23
	Sirolimus	4
Concomitant disorders (n)	Systemic hypertension	118
	Diabetes mellitus	60
	Dyslipidemia	65
	Hyperuricemia	15

^$^-Median (range); ^#^-The total number exceeds 146 as a few of them received a combination of immunosuppressive drugs.

**Table 2:** Dosing regimen of immunosuppressives in the study participants (N = 146).

Immunosuppressant Drugs	Dosing Regimen Per Day (n)	Median (Range) Dose/Day (mg)
Cyclosporine (n = 44)	25 mg OD (4)	100 (25–450)
	25 mg BD (2)	
	50 mg BD (16)	
	75 mg OD (1)	
	75 mg BD (2)	
	100 mg BD (5)	
	200 mg BD (3)	
	225 mg BD (1)	
	25-0-50 mg (1)	
	25-0-75 mg (1)	
	50-0-100 mg (1)	
	50-0-25 mg (3)	
	50-0-75 mg (1)	
	75-0-50 mg (1)	
	100-0-50 mg (1)	
	100-0-175 mg (1)	
Everolimus (n = 24)	0.25 mg OD (1)	1 (0.25–2)
	0.25 mg BD (1)	
	0.5 mg BD (9)	
	0.75 mg OD (1)	
	1 mg OD (2)	
	1 mg BD (5)	
	1.5 mg OD (1)	
	0.25-0-0.5 mg (1)	
	0.5-0-0.25 mg (1)	
	1-0-0.75 mg (1)	
	1-0-1 mg (1)	
Sirolimus (n = 3)	1 mg OD (3)	1
Tacrolimus (n = 77)	0.5 mg OD (1)	2 (0.5–9.5)
	0.5 mg BD (5)	
	1 mg OD (1)	
	1 mg BD (24)	
	1.5 mg BD (3)	
	2 mg OD (1)	
	2 mg BD (7)	
	3 mg BD (4)	
	3.5 mg BD (1)	
	1.5-0-1 mg (1)	
	1-0-0.5 mg (17)	
	1-0-1.5 mg (2)	
	2.5-0-2.5 mg (1)	
	2-0-1 mg (1)	
	2-0-1.5 mg (1)	
	2-0-2 mg (1)	
	2-0-2.5 mg (1)	
	3-0-2 mg (2)	
	3-0-2.5 mg (1)	
	4-0-3 mg (1)	
	5-0-4.5 mg (1)	

OD-Once daily; BD-Twice daily; Total numbers receiving immunosuppressive drugs exceed 146 as one each received everolimus followed by tacrolimus, and everolimus followed by cyclosporine.

**Table 3:** Distribution of SNPs in the study population.

SNPs		Numbers (%)
*ABCB1*	AA	26 (17.8)
	AG	80 (54.8)
	GG	40 (27.4)
POR*28	CC	66 (45.2)
	CT	62 (42.5)
	TT	18 (12.3)
*PPAR-alpha*	AA	7 (4.8)
	AG	45 (30.8)
	GG	94 (64.4)
CYP3A4*22	GG	120 (82.2)
	GA	20 (13.7)
	AA	6 (4.3)
CYP3A5*3	TT	8 (5.5)
	CT	46 (31.5)
	CC	92 (63)

**Table 4:** Analysis of overall dose-adjusted concentrations with the SNPs.

SNPs		Median (Range) Dose-Adjusted Concentrations	*p*-Values
CYP3A4*22	GG	2.8 (0.3–19.7)	0.8
	GA	2.9 (0.5–23.8)	
	AA	2.7 (0.9–5.2)	
CYP3A5*3	TT	2.5 (1.5–7.3)	0.4
	CT	2.2 (0.6–23.8)	
	CC	3.2 (0.3–19.7)	
*ABCB1*	AA	3.3 (0.7–7)	0.6
	AG	3.1 (0.3–23.8)	
	GG	2.3 (0.4–9.4)	
*PPAR-alpha*	AA	2.6 (1–5.8)	0.2
	AG	2.6 (0.3–7.3)	
	GG	3.1 (0.4–23.8)	
POR*28	CC	4 (0.6–12.5)	0.003 *
	CT	1.8 (0.3–19.7)	
	TT	3.9 (0.5–23.8)	
POR*28 activity	Normal	2.6 (0.3–19.8)	0.09
	Higher	3.9 (0.5–23.8)	

*-Statistically significant (*p* ≤ 0.05).

**Table 5:** Evaluation of mean drug concentrations with SNPs.

SNPs		Cyclosporine		Everolimus/Sirolimus		Tacrolimus	
		Median (Range) Concentrations	*p*-Values ^a,b^	Median (Range) Concentrations	*p*-Values ^a,b^	Median (Range) Concentrations	*p*-Values ^a,b^
CYP3A4*22	GG	103	0.2; 0.4	5.5 (3.6–12.1)	0.4; 0.4	7.6 (4.2–16.5)	0.2; 0.09
		(69.9–333.5)					
	GA	120.6		5.6 (5.2–10.1)		6.8 (4.8–8.5)	
		(102.1–162.1)					
	AA	90.5		Nil		7.7 (3.8–8.2)	
CYP3A5*3	TT	114.1	0.7; 0.6	5.9 (5.5–6.4)	0.8; 0.8	1.8 (1.5–5.3)	0.3; 0.3
	CT	116		5.5 (4.9–6.4)		2.7 (0.8–7)	
		(69.9–247.7)					
	CC	99.2		5.4 (3.6–12.1)		4.3 (1–19.7)	
		(83.3–333.5)					
*ABCB1*	AA	98.8	0.1; 0.7	5.3 (5–5.6)	0.2; 0.6	7.7 (5.3–10.7)	0.01*; 0.02 *
		(85.4–107.1)					
	AG	113.1		5.7 (3.9–12.1)		7.8 (4.8–16.5)	
		(84.3–247.7)					
	GG	97.2		4.7 (3.6–7.9)		6.7 (3.8–9.9)	
		(69.9–333.5)					
*PPAR-alpha*	AA	101.4	0.3; 0.2	5.5	0.5; 0.4	7.8 (4.8–8.7)	0.6; 0.4
		(98.4–104.4)					
	AG	115.6		5.3 (4.6–12.1)		7 (3.8–11)	
		(69.9–333.5)					
	GG	99 (83.3–247.7)		5.6 (3.6–10.1)		7.7 (4.2–16.5)	
POR*28	CC	98.8	0.4; 0.9	5.6 (3.9–12.1)	0.5; 0.3	7.8 (4.2–13.5)	0.8; 0.9
		(83.3–333.5)					
	CT	105.6		5.3 (4.9–10)		7.1 (3.8–16.5)	
		(69.9–204.3)					
	TT	96.7		5.1 (3.6–6)		7.7 (4.7–10.7)	
		(85.4–114.1)					
POR*28 activity	Normal	103 (41–69.8)	NA	5.5 (3.9–12.1)	NA	7.5 (3.8–16.5)	NA
	Higher	96.7 (85.4–114)		5.1 (3.6–6)		7.7 (4.7–10.7)	

a-for differences between the genotypes; b-for trend analysis; *-Statistically significant (*p* ≤ 0.05); NA-Not assessable due to smaller numbers with higher levels of activity.

**Table 6:** Evaluation of dose-adjusted concentrations with SNPs.

SNPs		Cyclosporine		Everolimus/Sirolimus		Tacrolimus	
		Median (range) Dose-Adjusted Concentrations	*p*-Values ^a,b^	Median (range) Dose-Adjusted Concentrations	*p*-Values ^a,b^	Median (Range) Dose-Adjusted Concentrations	*p*-Values ^a,b^
CYP3A4*22	GG	1.1 (0.3–4.6)	0.6; 0.3	5.1 (2.7–7.9)	0.7; 0.7	3.6 (0.8–19.7)	0.2; 0.08
	GA	0.7 (0.5–1.6)		5.2 (2.6–23.8)		2.9 (1–4.3)	
	AA	0.9		Nil		3.4 (1.3–5.2)	
CYP3A5*3	TT	1.5	0.2; 0.2	6.8 (6.4–7.3)	0.2; 0.9	1.8 (1.5–5.3)	0.05 *; 0.2
	CT	1.2 (0.6–4.6)		5.7 (4.9–23.8)		2.7 (0.8–7)	
	CC	1 (0.3–4.1)		4.9 (2.6–7.9)		4.3 (1–19.7)	
*ABCB1*	AA	1.1 (0.7–4)	0.5; 0.9	5.1 (2.8–5.3)	0.3; 0.8	3.5 (1.3–7)	0.7; 0.5
	AG	1 (0.3–4.6)		6.1 (2.7–23.8)		3.7 (0.8–19.7)	
	GG	1.2 (0.4–4.1)		4.7 (2.6–7.9)		2.7 (0.8–9.4)	
*PPAR-alpha*	AA	1.2 (1–1.4)	0.09; 0.08	3.7	0.6; 0.7	2.9 (1–5.8)	0.3; 0.09
	AG	0.8 (0.3–2.2)		5.3 (4.6–7.3)		3.4 (1–5.3)	
	GG	1.2 (0.4–4.6)		5 (2.6–23.8)		3.6 (0.8–19.7)	
POR*28	CC	1.1 (0.6–4.6)	0.05 *; 0.02 *	5.5 (2.8–7.9)	0.8; 0.5	4.6 (1–12.5)	0.007 *; 0.5
	CT	0.9 (0.3–4.1)		5.3 (2.6–7.3)		2.6 (0.8–19.7)	
	TT	0.7 (0.5–1.5)		4.2 (2.7–23.8)		4.9 (2.4–7)	
POR*28 activity	Normal	1 (0.3–4.6)	NA	5.3 (2.6–7.9)	NA	3.2 (0.8–19.7)	NA
	Higher	0.7 (0.5–1.5)		4.2 (2.7–23.8)		4.9 (2.4–7)	

a-for differences between the genotypes; b-for trend analysis; *-Statistically significant (*p* ≤ 0.05); NA-Not assessable due to smaller numbers with higher levels of activity.

**Table 7:** Comparison of SNPs with the time spent in the therapeutic range.

SNPs		Cyclosporine		Everolimus/Sirolimus		Tacrolimus	
		Median (Range) Time Spent in Therapeutic Range (%)	*p*-Values ^a^	Median (Range) Time Spent in Therapeutic Range (%)	*p*-Values ^a^	Median (Range) Time Spent in Therapeutic Range (%)	*p*-Values ^a^
CYP3A4*22	GG	50.2 (9–81)	0.2	97 (52–100)	0.3	90.5 (24.4–100)	0.8
	GA	63.8 (55–70)		99 (44–100)		75.8 (32.6–100)	
	AA	49.4		NA		87.1 (24.1–100)	
CYP3A5*3	TT	49.5	0.8	92.6 (85.2–100)	0.7	91.8 (37.6–96.6)	0.7
	CT	54.7 (17–85)		99.6 (96.1–100)		90.1 (37.6–100)	
	CC	51 (9–81)		96.3 (44–100)		85.8 (24.1–100)	
*ABCB1*	AA	46.4 (35–52)	0.04 *	100 (99.2–100)	0.05 *	87.3 (38.7–99.1)	0.2
	AG	56.5 (17–81)		96.6 (44–100)		93.6 (31.1–100)	
	GG	40.5 (9–66)		75.4 (60–100)		85.6 (24.1–100)	
*PPAR-alpha*	AA	55 (51–59)	0.3	97	0.4	91.7 (89.6–96.5)	0.8
	AG	55.3 (35–70)		100 (60–100)		91.3 (24.1–100)	
	GG	49.2 (9–81)		95.8 (44–100)		87 (24.4–100)	
POR*28	CC	50.2 (9–81)	0.6	93.9 (44–100)	0.08	91.7 (24.4–100)	0.4
	CT	56.5 (17–70)		100 (93–100)		85.8 (24.1–100)	
	TT	47.6 (46–50)		95.8 (69.9–97)		87.6 (28.9–99.1)	
POR*28 activity	Normal	51.4 (9–81)	0.5	99.6 (44–100)	0.07	90.5 (24.1–100)	0.6
	Higher	47.6 (46–50)		95.8 (69.9–97)		87.6 (28.9–99.1)	

a-for differences between the genotypes *-Statistically significant (*p* ≤ 0.05).

**Table 8:** Laboratory-related adverse events amongst the study participants.

Adverse Events	Number of Patients	Cyclosporine	Everolimus/ Sirolimus	Tacrolimus	*p*-Values
Elevated serum creatinine (number of events = 49)	Number of patients	15 (37.5%)	6 (24%)	28 (37.3%)	0.4
	Total number of patients evaluated	40	25	75	
Hyperbilirubinemia (number of events = 20)	Number of patients	9 (45%)	1 (7.7%)	10 (25%)	0.06
	Total number of patients evaluated	20	13	40	
Elevated GGT (number of events = 34)	Number of patients	15 (35.7%)	7 (28%)	12 (16.2%)	0.05 *
	Total number of patients evaluated	42	25	74	
Hypercholesterolemia (number of events = 92)	Number of patients	28 (65.1%)	20 (80%)	44 (60.3%)	0.2
	Total number of patients evaluated	43	25	73	
Hypertriglyceridemia (number of events = 116)	Number of patients	36 (83.7%)	20 (80%)	60 (62.5%)	0.8
	Total number of patients evaluated	4	25	96	

GGT—Gamma glutamyl transferase; NA-Not analyzable; *-Statistically significant (*p* ≤ 0.05).

## Data Availability

The data is available with the corresponding author and shall be shared upon a reasonable request.
